# Cooling combined with hyperoxic CO_2_ anesthesia is effective in improving the air exposure duration of tilapia

**DOI:** 10.1038/s41598-017-14212-3

**Published:** 2017-10-25

**Authors:** Wei-Liang Guan, Mou-Ming Zhao, Tian-Tian Liu, Xing Fan, De-Wei Chen

**Affiliations:** Department of Food Science, Guangxi University, Nanning, Guangxi 530004 China

## Abstract

Tilapia were subjected to cooling (CO, a stepwise reduction in temperature from 30 °C to 15 °C), anesthesia (AN, anesthetized by hyperoxic carbon dioxide), air exposure (AE, exposed to air) and cold tolerance (CT, in 15 °C water) treatments, and the physiological responses were determined after the treatments. CO followed by AN treatment for tilapia could meet the criteria of an ideal anesthetic. Fish were deeply sedated within 69 s, completely anesthetized within 276 s and recovered within 308 s without any mortality. The stress responses induced by the CO&AN treatment were mild, whereas they were consistently increased in the AE treatment. Furthermore, the AE treatment caused tissue damage. The AE duration was significantly improved by CO&AN treatment, and the survival time of the CO&AE, AN&AE and CO&AN&AE treatments were 313 min, 351 min and 561 min, respectively, in the laboratory experiments, whereas the survival rate of the CO&AN&AE treatment group after 240-min air exposure was 95.2% in the pilot test. It appeared that cooling followed by hyperoxic CO_2_ anesthesia would be suitable for handling tilapia in a short-time air exposure procedure.

## Introduction

Air exposure can induce acute stress in fish, and asphyxia is the major stressor caused by air exposure, which induces serious physiological disturbances in fish, including variations of cortisol, glucose and lactate in plasma, hematological responses, visceral organ damage and, finally, mortality^1^. Temperature and stress during air exposure are the major factors linked to survival rate. Fish have an optimal temperature range, in which no physiological disturbances occur, and the metabolic rate is proportional to temperature within this range^2^. A higher survival rate was found in bluegill after air exposure at a low temperature compared with the treatment performed at a high temperature^3^. Therefore, regulation by reducing stress and temperature could be a method for improving the air exposure duration of fish.

Anesthetics are usually used for sedation in fishery operations^4,5^. In our previous study^6^, tilapia were anesthetized by 3-aminobenzoic acid ethyl ester methanesulfonate (MS-222), and the results showed that anesthetized fish had a prolonged air exposure duration. Although MS-222 remains the only fish anesthetic that has been approved by the Food and Drug Administration (FDA) in the United States, fish treated with MS-222 are not edible until after a 21-day withdrawal period^7^. Therefore, the use of MS-222 in food aquatic products is limited. However, a CO_2_ water solution was assessed to be an effective anesthetic for aquatic animals, and CO_2_ is safe and is not a risk to humans^8^. Although this has advantages, the stress caused by CO_2_ cannot be neglected^7^. Sandblom *et al*.^9^ anesthetized Arctic char (*Salvelinus alpinus L*.) and Kugino *et al*.^10^ anesthetized chicken grunts with oxygenated CO_2_, and the results suggested that hyperoxic CO_2_ was effective and even essential in anesthesia and reducing stress.

The metabolic rate of fish decreases with decreasing temperatures, and oxygen consumption is lowered in cold adapted fish^2^. Although live chilling is a widely used method for fish sedation^11^, the rapid decrease in temperature can result in a number of physiological, behavioral and fitness consequences for fish, termed “cold shock”^12^. The stress response is proportional to the magnitude and the rate of decreased temperature^12^. A stepwise reduction in temperature is necessary to lower the stress response^13^. Tilapia are mostly farmed in tropical and subtropical regions, and the capture temperature is between 25 and 28 °C^14^, which indicates that tilapia are usually exposed to a high temperature in the fishery. Therefore, cooling via a stepwise reduction in temperature from ambient temperature to approximately the lethal temperature for tilapia may be necessary for lowering the metabolic rate and stress response in tilapia.

The objective of this study was to improve the air exposure duration of tilapia by combining cooling with hyperoxic CO_2_ anesthesia. The results from this study are useful for developing a new strategy of handling tilapia in a short-time air exposure procedure during fish processing.

## Results

### Behavioral responses to anesthesia

**Figure 1:**
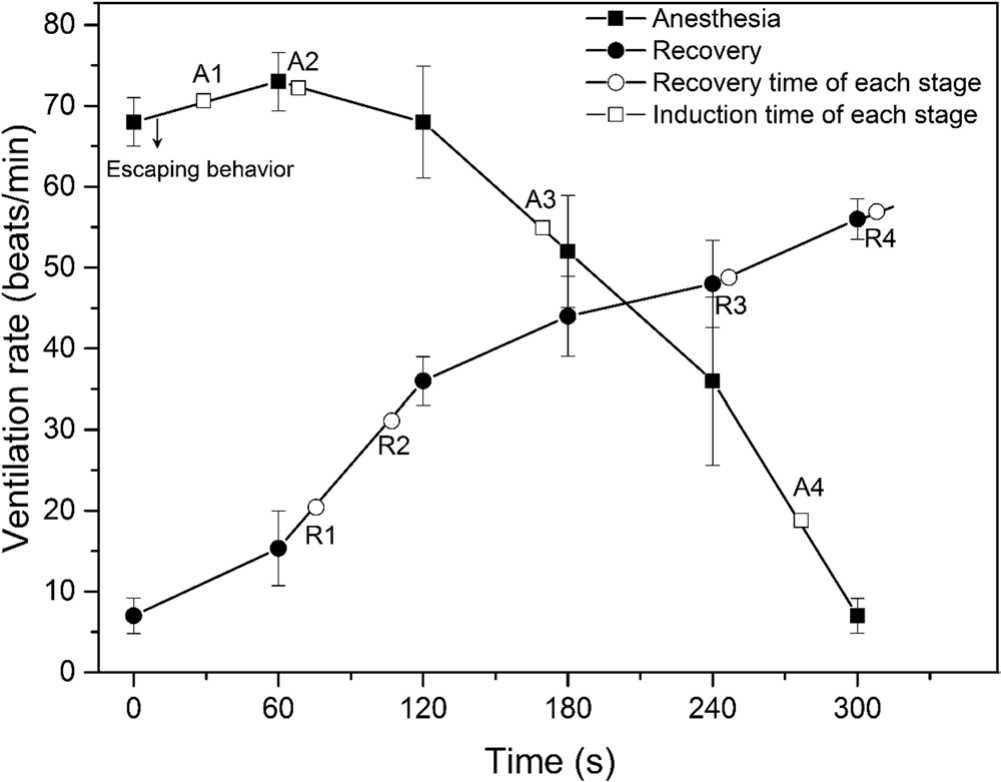
Ventilation rates and time points of each stage in anesthesia and recovery. This experiment was carried out in triplicate. All values are means ± SD (n = 15, 5 per replicate).

Fish showed adverse behavior, such as escaping at the beginning (within 30 s), and then, they calmed down quickly. All of the subjected fish were deeply sedated within 69 s, completely anesthetized within 276 s and recovered within 308 s without any mortality. In the anesthesia process, hyperventilation was noted at the beginning and then decreased gradually to 7 beats/min in the end. In the recovery process, ventilation rates elevated gradually from 7 beats/min at 0 s to 56 beats/min at 300 s (Fig. 1).

### Plasma parameters

In the anesthesia experiment, the primary response (cortisol) significantly elevated 10 min after anesthesia, persistently increased within 30 min of recovery, and decreased to baseline until 90 min of recovery. For secondary responses, only HCT increased after anesthesia, but elevations in glucose and lactate were noted at 30 min of recovery. Afterward, HCT and glucose returned to basal values after 90 min of recovery (Table 1).

**Table 1:** Hematological responses to anesthesia and air exposure experiment in tilapia.

Treatment		Cortisol (nmol/L)	Glucose (mmol/L)	Lactate (mmol/L)	AST (U/L)	ALT (U/L)	RBC (10^12^ cells/L)	HCT (%)
Anesthesia experiment	10 min anesthesia	73.3 ± 8.4^d^	8.5 ± 0.5^d^	1.2 ± 0.2^d^	ND	ND	ND	33.7 ± 1.2^b^
	30 min recovery	104.3 ± 14.7^b^	14.9 ± 1.4^b^	2.4 ± 0.3^bc^	ND	ND	ND	31.7 ± 1.5^bc^
	90 min recovery	47.2 ± 6.4^e^	8.9 ± 0.5^d^	2.7 ± 0.5^ab^	ND	ND	ND	30.0 ± 1.0^bc^
	Rest	53.5 ± 7.3^e^	9.5 ± 1.6^d^	0.9 ± 0.3^d^	82.0 ± 11.0^b^	33.0 ± 10.8^b^	2.8 ± 0.1^b^	28.5 ± 1.0 ^cd^
Air exposure experiment	CO	77.1 ± 10.7 ^cd^	14.7 ± 1.3^b^	2.0 ± 1.7^bcd^	105.3 ± 8.0^b^	30.7 ± 10.8^b^	2.3 ± 0.6^b^	25.3 ± 6.4^d^
	CO&AN&CT	93.0 ± 6.6^bc^	12.8 ± 0.5^bc^	0.6 ± 0.3^d^	114.3 ± 12.1^b^	41.3 ± 16.3^b^	2.8 ± 0.1^b^	31.3 ± 1.2^bc^
	CO&AN&AE	186.0 ± 8.6^a^	21.6 ± 0.9^a^	4.7 ± 2.0^a^	224.3 ± 23.7^a^	128.0 ± 30.0^a^	3.6 ± 0.1^a^	39.7 ± 2.3^a^

10 min anesthesia, 30 min recovery and 90 min recovery refer to the anesthesia experiment; CO, CO&AN&CT and CO&AN&AE refer to the air exposure experiment. CO = cooling, CO&AN&CT = cooling, anesthesia and cold tolerance control, CO&AN&AE = cooling, anesthesia and air exposure treatment, ND = not detected. Values are means ± SD (n = 10, 5 per replicate). Different letters indicate significant differences (*P* < 0.05) between treatments. Rest values were taken from fish without any treatment (30 °C).

Cortisol was elevated in the CO&AN&CT treatment, and moreover, it increased 3.5-fold in the CO&AN&AE treatment. The maximums of glucose, AST, ALT, RBCs and HCT were all noted in the CO&AN&AE treatment. Lactate significantly increased after the CO treatment and then returned to the basal value after the CO&AN&CT treatment, whereas it exhibited a 5-fold increase in the CO&AN&AE treatment (Table 1).

### Survival rates during air exposure

The survival rates in air exposure for each laboratory treatment are exhibited in Table 2. Mortality occurred after 480 min in the CO&AN&AE treatment, and a large portion of deaths did not occur until 540 min after air exposure. However, mortality occurred after 240 min of air exposure in both the CO&AE and the AN&AE treatments, and no living fish were found after 420 min and 480 min, respectively.

**Table 2:** Survival rates and survival time of each laboratory treatment.

Treatments	Survival rates at different times (%)									Survival time (min)
Time (min)	120	240	300	360	420	480	540	600	660	
CO&AN&AE	100 ± 0	100 ± 0	100 ± 0	100 ± 0	100 ± 0	93 ± 12	67 ± 12	20 ± 0	7 ± 12	561 ± 57^a^
CO&AE	100 ± 0	93 ± 12	67 ± 12	40 ± 20	0	0	0	0	0	313 ± 57^b^
AN&AE	100 ± 0	93 ± 12	60 ± 0	40 ± 12	33 ± 12	0	0	0	0	351 ± 79^b^

CO&AN&AE = cooling, anesthesia and air exposure treatment, CO&AE = cooling and air exposure control, AN&AE = anesthesia and air exposure control. All treatments were run in triplicate (n = 15, 5 per replicate). Values are presented as the mean ± SD. Significant differences (*P* < 0.05) between the survival times of each treatment are indicated by different letters.

In the pilot test, after the 240-min air exposure procedure, 452 living fish were found in the CO&AN&AE treatment group, and the survival rate was 95.2%. By contrast, no survivors were found in the control group.

## Discussion

Tilapia could be completely anesthetized by hyperoxic CO_2_ and recovered without any mortality in the present study, which indicated that hyperoxic CO_2_ anesthesia was efficient for tilapia. An effective anesthetic should induce anesthesia within 3 min and allow recovery within 5 min^7^. The induction time (276 s) and recovery time (308 s) were both longer than the criteria. However, for some operations in aquaculture, fish under deep sedation (A2, 69 s in the present study) should be sufficient. If fish were excessively anesthetized by hyperoxic CO_2_, hypercapnia and a decrease in blood pH could be induced and could result in direct or delayed mortality^8,15^, which would surely compromise the survival rate during air exposure. Furthermore, in our previous study, the highest survival rate of the anesthetized tilapia was noted when fish were anesthetized in the A2 stage^6^. Therefore, in the present experiment, fish were merely anesthetized into a state of deep sedation (A2) to minimize stress and avoid hypercapnia. In comparison with other reports^9,11^, the present study seemed to be more effective, and the difference might be due to the environment (such as temperature, ions, and water) and species. The temperature was 30 °C for tilapia but 10 °C for Arctic char. The lower temperature meant a higher CO_2_ concentration and a state of lower metabolic rate, which caused a shorter induction time and a longer recovery time. With the high concentration of ions in the marine environment, the solubility of CO_2_ is reduced; thus, it is more difficult to achieve sedative concentrations of CO_2_ in salt water than in fresh water^11^. Additionally, CO_2_ excretion occurs more readily in the marine environment, making it more difficult for hypercapnia to be induced in marine species. Moreover, tilapia have many positive attributes (such as tolerance for poor water quality and tolerance to handling and crowding)^16^; thus, tilapia were effectively anesthetized by the hyperoxic CO_2_ exposure treatment.

The ventilation response of tilapia was in agreement with that of Arctic char exposed to hyperoxic CO_2_^9^, and a slight elevation in the ventilation rate was noted at the beginning of anesthesia exposure (Fig. 1). High concentrations of CO_2_ inhibited the combination of O_2_ and hemoglobin, so the ventilation rate of the conscious fish increased for O_2_ intake. The ventilation rate was positively correlated with oxygen consumption and the metabolic rate^17^. According to the ventilation results, the metabolic rate of anesthetized tilapia decreased. The physiological responses of anesthesia suggested that hyperoxic CO_2_ seemed to be an ideal anesthetic for tilapia.

Cortisol, glucose and lactate are common indicators of stress^18^. Cortisol is primarily associated with eliciting metabolic shifts in accordance with escape responses^19^. The increasing of glucose was caused by glycogenolysis and/or gluconeogenesis, which were/was elevated by cortisol. Lactate is an important product of glucose and was produced via anaerobic metabolism during heightened swimming activity^19,20^. In the AN treatment, cortisol, glucose and lactate reached their peak values at 30 min during recovery and returned to basal values at 90 min post-recovery (except for lactate). These results were in agreement with those of Sandblom *et al*.^9^, who detected the maximum at 30 min post-recovery in Arctic char following 10 min of hyperoxic CO_2_ exposure. The maximum of cortisol in Arctic char was approximately 245 nmol/L, which was 4-fold its basal value, and the elevated cortisol level was higher in Arctic char than that in tilapia of the present experiment. In the present experiment, the stress responses of tilapia caused by hyperoxic CO_2_ exposure resulted in changes in cortisol, glucose and lactate. However, this stress was mild and temporary, and tilapia was suitable for hyperoxic CO_2_ exposure.

No behavioral disturbances were observed in the CO treatment, which suggested that brain functions of tilapia were not compromised with the temperature reduction. The indicators of hormonal and metabolic stress responses increased slightly after the CO treatment, and the glucose and lactate levels decreased after the CT treatment, which indicated that the slight stress caused by CO was relieved during CT. This result was in agreement with that of Chen *et al*.^21^, who challenged tilapia by temperature reduction from 25 °C to 12 °C within 30 min. The results of the physiological responses demonstrated that the rate of temperature drop in the present experiment did not induce “cold shock” in tilapia.

The CO&AN&CT treatment was deliberately set to be identically to the CO&AN&AE treatment, except for holding in water as a sham control group to isolate and analyze the stress caused by AE. The present data illustrated that the AE treatment elicited a much more severe stress response compared with the CT treatment, as most parameters dramatically changed after the AE treatment (Table 1). Similar results were also found in Largemouth Bronze Gudgeon (*Coreius guichenoti*)^22^ and cobia^20^ challenged to air exposure for 1 min. Interestingly, lactate returned to its basal value in the CT treatment, whereas it subsequently increased in the AE treatment, because lactate caused by CO generally lasted short term and recovered over a short period^12^. Therefore, lactate dissipated from the bloodstream during CT, while it was produced during AE. AST and ALT are intracellular enzymes, and they are indicators of cell tissue damage, as their functions are restricted to the intracellular space, and they are only released in the blood stream upon cell damage or death^23^. In the present experiment, increases in AST and ALT were induced by AE rather than temperature drop because there were no significant changes in the CO&AN&CT treatment, while they significantly increased in the CO&AN&AE treatment. Similar results were also noted in the common carp^23^ and Largemouth Bronze Gudgeon^22^ after air exposure.

Fish suffered acute anoxia during air exposure. To cope with the respiratory stress, some species of teleost, such as bonefish^24^, loricariid fish^25^ and the common carp^23^, increased their oxygen-carrying capacity, which was accomplished by releasing stored red blood cells (RBCs), accelerating the maturation of circulating immature RBCs and/or stimulating erythropoiesis^26,27^. In the present experiment, RBCs significantly increased after AE treatment, which suggested that new RBCs were compensated during AE. Releasing additional RBCs and erythrocytic swelling could induce an elevation in HCT, and HCT was another common stress indicator for fish^18^. The HCT of tilapia was significantly elevated by air exposure (Table 1) to increase the capacity of oxygen binding.

Obviously, both cooling and anesthesia played important roles in improving air exposure duration and increasing the survival rate of air exposure. Physiological responses during air exposure resulted in elevated cortisol, which was a predictor of mortality^1^. Although the cortisol responses were different among species and experiment protocols, the value of cortisol in tilapia (186 nmol/L, Table 1) was less than those in other species after air exposure, such as 883 nmol/L in Largemouth Bronze Gudgeon^22^ and 634.8 nmol/L in cobia^20^. The limited cortisol response might explain why anesthetized tilapia had prolonged air exposure duration.

When fish were out of water, high temperatures accelerated tissue energy consumption^28^. Mortality occurred due to asphyxia, depletion of tissue energy and severe physiological disturbances caused by air exposure^1^. Temperature was highly related to the survival rate of air exposure; for example, little skate (*Leucoraja erinacea*) exposed to air for 50 min, both in winter (1 °C) and summer (27 °C), had a mortality rate of 27% in the winter group and 100% in the summer group^29^. In the present study, cooling could lower the energy consumption of fish; thus, cooled tilapia exhibited improved air exposure duration. Although the AN&AE treatment was also performed at a low temperature, the large magnitude of temperature reduction, which induced additional stress, impaired the duration of air exposure. Additionally, the increase in RBCs of tilapia during air exposure improved the capacity of oxygen binding, which might be another reason that tilapia had a much longer air exposure duration than other species.

To verify whether cooling followed by hyperoxic CO_2_ anesthesia had good sedative effects and prolonged the survival time during air exposure, a pilot test was performed. In the pilot test, although the survival rate was compromised compared with the laboratory experiment, the survival rate was improved by cooling combined with anesthesia. The different results between the laboratory experiment and the pilot test were primarily caused by the additional stress during handling and air exposure, including the retention prior to capture, the sunniness in capture and the handling process, and the noise and shaking during transport. In further research, to achieve a higher survival rate or a longer air exposure duration, cooling would be prolonged prior to anesthesia, and handling stress would be lowered to avoid hypercapnia.

It is necessary to keep fish sedated when they are exposed to air for a short time (approximately 10–240 min, depending on the distance of transport and process scale) during the transport and processing procedure (grading, weighing, slaughter, *etc*.). The results of the pilot test implied that cooling followed by hyperoxic CO_2_ anesthesia had good sedative effects and prolonged the survival time during air exposure in the present study. The use of CO_2_ anesthesia in bony fish is controversial, and some people are strongly against it. However, in our present experiment, cooling followed by hyperoxic CO_2_ anesthesia for tilapia could meet the criteria of an ideal anesthetic: safe for fish and consumers; inexpensive; easy to handle; and short induction time and recovery time^7^. Therefore, cooling followed by hyperoxic CO_2_ anesthesia could be applied in handling tilapia in a short-time air exposure procedure during fish processing.

## Methods

### Animal holding and general experimental protocol

All of the experiments were performed according to the guide of the National Research Council (US) Committee for the care and use of laboratory animals and the ethical permit approved by the Animal Ethics Committee of Guangxi University. Farmed tilapia (Oreochromis niloticus) with weights ranging from 500–550 g were purchased from a local freshwater farm and transported with a tank full of air aerated freshwater (no anesthetics) to the laboratory within 20 min at a density of 250 g/L. They were placed in the laboratory for two days before the experiment, in continuously aerated 50 L tanks, and the stocking density was 20 fish per tank (30 ± 2 °C, pH 6.8–7.2, dissolved oxygen 9 mg/L).

Behavioral response experiments were performed prior to hematological response experiments, and the pilot test was performed last. Specifically, behavioral responses of anesthesia (anesthesia time, recovery time, ventilation rate, *etc*.) and air exposure (survival rate) were determined. Afterward, these experiments were performed once again, and blood samples were taken at the settled time for hematological parameter detection.

### Anesthetic preparation

The hyperoxic CO_2_ saturated water solution was prepared by bubbling CO_2_/O_2_ (1.0 L/min) mixture into 20 L water (30 °C) held by the anesthesia tank with a capacity of 25 L. The pH value was determined by a pH meter (Leici PHS-3E, China), and dissolved oxygen and carbon dioxide were measured by chemical methods described by Montgomery *et al*.^30^ and Pauss *et al*.^31^, respectively. The gas flow was decreased to 0.2 L/min until the pH reached 5.6 (dissolved CO_2_ was 320 mg/L) and dissolved oxygen concentrations reached 14 mg/L, which meant that both dissolved CO_2_ and O_2_ reached the saturated concentration. Another same-sized tank with oxygenated fresh water (dissolved oxygen was 9 mg/L, 30 °C) was required for fish recovery.

### Anesthesia efficacy

Five rested fish were randomly netted from the resting tank and transferred to the anesthesia tank at the same time. Fish were transferred into the recovery tank immediately when they were completely anesthetized (A4). Behaviors were observed to define different induction stages and recovery stages; operculum movement was checked visually to determine respiratory frequency. Four anesthesia stages and four recovery stages were based on fish behavior according to Erikson’s description^32^ with slight modification (Table 3). The time from exposure to the beginning of each stage (both anesthesia and recovery) was determined. The trial was run in triplicate.

**Table 3:** Behavioral changes at various stages of anesthesia and recovery.

Stage	Description	Behavior
A1	Light sedation	Partial loss of reaction to external stimuli
A2	Deep sedation	Partial loss of equilibrium, no reaction to external stimuli
A3	Anesthesia	Fish turns over, loses swimming ability
A4	Complete anesthesia	No external reaction, no operculum movement
R1	Incomplete previous stunning	Occasional opercular motions, fish is lying on bottom
R2	Incomplete previous stunning	Respiration recover, fish still turns over
R3	Escape behavior	Fish regains partial equilibrium
R4	Normal	Total recovery of equilibrium, normal swimming

### Cooling, anesthesia and air exposure

In the present treatment, fish were subjected to cooling (CO), then anesthesia (AN), and finally air exposure (AE) (CO&AN&AE treatment). In the CO treatment, fish were subjected to a stepwise reduction in temperature from 30 °C to 15 °C, at a rate of 3.5 °C/h, by adding slurry ice. The change of temperature was monitored by a temperature meter and a timer. The final cooling temperature was set to 15 °C because the lethal temperature of tilapia is 12 °C^33^. As soon as the temperature reached the final cooling temperature, fish were transferred to the anesthesia tank filled with hyperoxic CO_2_ water at 15 °C for anesthesia exposure (AN treatment). As soon as fish were anesthetized into stage 2 (A2), they were removed to a ventilated case for the air exposure experiment (AE treatment) until death. The cases were kept at 15 °C. Fish were detached from each other, and fish were sprayed with constant-temperature water (15 ± 1 °C) every 10 min during air exposure until all fish died.

**Figure 2:**
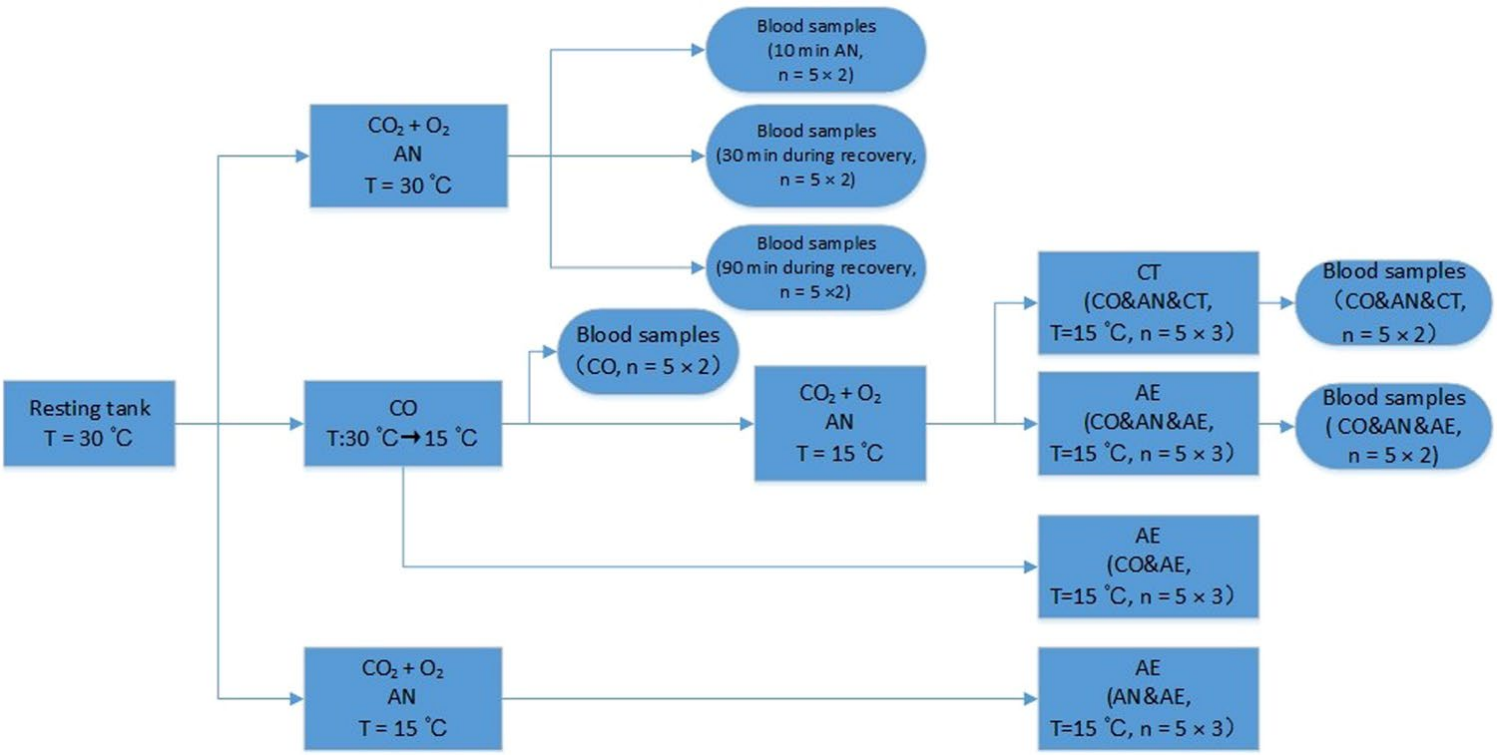
Illustration of the experimental protocol. Blood sample experiments were performed independently. Behavioral response experiments (anesthesia response, survival rate of air exposure, *etc*.) were performed prior to hematological response experiments, all behavioral response experiments were triplicated, and 5 fish were subjected in each replicate (5 × 3); all hematological response experiments were duplicated, and 5 fish were subjected in each replicate (5 × 2).

Three control groups were performed as cooling and air exposure control (CO&AE), anesthesia and air exposure control (AN&AE), and cooling, anesthesia and cold tolerance control (CO&AN&CT). 1) The CO&AE fish were subjected to cooling but without anesthesia and then air exposure; 2) the AN&AE fish were anesthetized but without cooling and then subjected to air exposure; and 3) the CO&AN&CT fish were subjected to the same as the CO&AN&AE treatment but instead of being exposed to air were returned to water at 15 °C (Fig. 2).

When the mouths and gills stopped moving for more than 1 min, the fish were judged to be dead^26^, and the death check was carried out continuously. The time from the beginning of the air exposure to the individual fish death was recorded. All treatments were performed in triplicate, and five fish were used for each treatment per time.

### Blood sampling

Blood samples were taken as shown in Fig. 2. In the anesthesia experiment, fish were sampled at 10 min after anesthesia and 30 min and 90 min during the recovery period. Blood samples were also taken at the end of cooling and 7 h after cold tolerance and air exposure. Each treatment was performed and replicated as described above, five subjected and rested (control, rested two days after being transported to the laboratory) live fish were sampled, and individual fish were sampled only once. Blood samples of the live fish were taken from the caudal vessel by a vacuum blood tube equipped with a needle pipe. All blood samples were collected within 3 min. Blood samples were taken for separate analyses; one used a heparinized vacuum blood tube for hematocrit (HCT) and total red blood cell count (RBC) determination by a blood cell analyzer (Mindray BC-2600, China), and the other one used no additive vacuum blood tube for plasma collection. Whole blood was centrifuged (2000 g, 5 min), and then, plasma was removed and stored at −80 °C. Plasma was collected for cortisol, glucose, lactate, aspartate aminotransferase (AST) and alanine aminotransferase (ALT) analysis. A radioimmunoassay (RIA) was used for cortisol determination by an Immunoassay Analyzer (DPC Immulite2000, USA); glucose, lactate, AST and ALT were determined by an Automatic Biochemistry Analyzer (Toshiba TBA-120FR, Japan).

### Pilot test

The pilot test was performed at a fishpond, for which the water temperature was 26 °C, in the suburb of Nanning, and the weather was sunny. In total, 500 fish (length = 30.6 cm, weight = 711.4 g, on average) were retained in a small area by a mesh net prior to capture. Twenty fish were captured once by a net, put into a tank (50 × 60 × 50, capacity of 150 L) filled with hyperoxic CO_2_ water (20 °C) and held there 1 min for simplified cooling and anesthesia. Subsequently, they were netted to a ventilated case (80 × 60 × 30 cm) and then loaded into a van container, which kept constant temperature (18 °C) and gas exchange. A total of 475 tilapia were treated by the present method, while 25 were directly netted to the case set as the control group. The loading density was 250 kg/m^3^. Death check in the pilot test was carried out at the time of 240 min after air exposure.

The cooling process was simplified in the pilot test. A similar temperature reduction of 6 °C (from 25 °C to 19 °C) occurred in tilapia within 15 min, while no significant change in cortisol was noted^21^, which could indicate that the stress caused by temperature reduction in the pilot test was mild.

### Statistics

Values were presented as the mean ± SD, and SPSS19.0 for Windows (SPSS Inc., Chicago, Illinois, USA) was used for statistical analysis. Data were first tested for normality (Shapiro-Wilk test) and homogeneity (levene test), and all data passed both tests. The significance of any differences between the groups or the impact of treatment was determined using one-factor analysis of variance (ANOVA), followed by a post hoc Tukey’s test. Differences at the level of *P* < 0.05 were considered to be statistically significant.
